# Relapsed papillary urothelial neoplasm of low malignant potential (PUNLMP) of the young age: a case report and a review of the literature

**DOI:** 10.1186/s12894-019-0469-1

**Published:** 2019-05-09

**Authors:** Palma Maurizi, Michele Antonio Capozza, Silvia Triarico, Maria Luisa Perrotta, Vito Briganti, Antonio Ruggiero

**Affiliations:** 1 Pediatric Oncology Unit, Foundation “A. Gemelli” Hospital IRCCS - Catholic University of Sacred Hearth, Largo A. Gemelli 8, 00168 Rome, Italy; 2 Pediatric Surgery Unit, San Camillo Forlanini Hospital, Rome, Italy

**Keywords:** Papillary urothelial bladder neoplasm, Low grade of malignancy, Relapsed disease, Intrabbladder chemotherapy

## Abstract

**Background:**

Papillary Urothelial Neoplasm of Low Malignant Potential (PUNLMP) are exceptionally rare in the first decade of life (mostly if multifocal) and there is a lack of standardized recommendations for the pediatric age.

**Case presentation:**

We describe the case of a 9-year-old boy with a diagnosis of PUNLMP, who underwent to cystoscopic lesion removal and later to endoscopic lesion removal and intra-bladder Mitomycin-c (MMC) instillations for relapsed disease. Follow-up investigations at five years showed disease negativity.

**Conclusions:**

Intra-bladder MMC instillation may allow obtaining the complete remission with bladder-sparing for paediatric patients with a high-risk relapsed PUNLMP.

## Background

Urothelial bladder neoplasms are extremely rare in the first decades of life, with an incidence of 0.1–0.4% and less than 35 cases described in children below ten years of age [1–3].

The most typical form of young age is the Papillary Urothelial Neoplasm of Low Malignant Potential (PUNLMP), which is biologically indolent and low tumour grading and staging. Survival at five years of age is reported at about 95% [4].

Multifocal forms are sporadic and there are currently no cases described in the literature in children under ten years. Although these tumours are mostly superficial and low grading, it is difficult to clearly define the aetiology, the invasive potential, the optimal treatment and expected survival [5].

## Case presentation

We describe the case of an otherwise healthy 9-y-old boy with a bladder neoplasm, whose clinical history started a year before years with macroscopic haematuria.

The cystoscopy showed the presence at the right ureteral meatus of papillomatous structure (of about 2 cm of diameters), which was entirely removed through transurethral resection (TUR). The histology revealed “urothelial papillary neoplasia with a low degree of malignancy, without infiltration of the sub-epithelial connective tissue”, according to the 2004 WHO/ISUP (World Health Organization/International Society of Urological Pathology classification.

Then, the patient underwent a six-monthly follow-up, with regular clinical and radiologic screening.

**Fig. 1:**
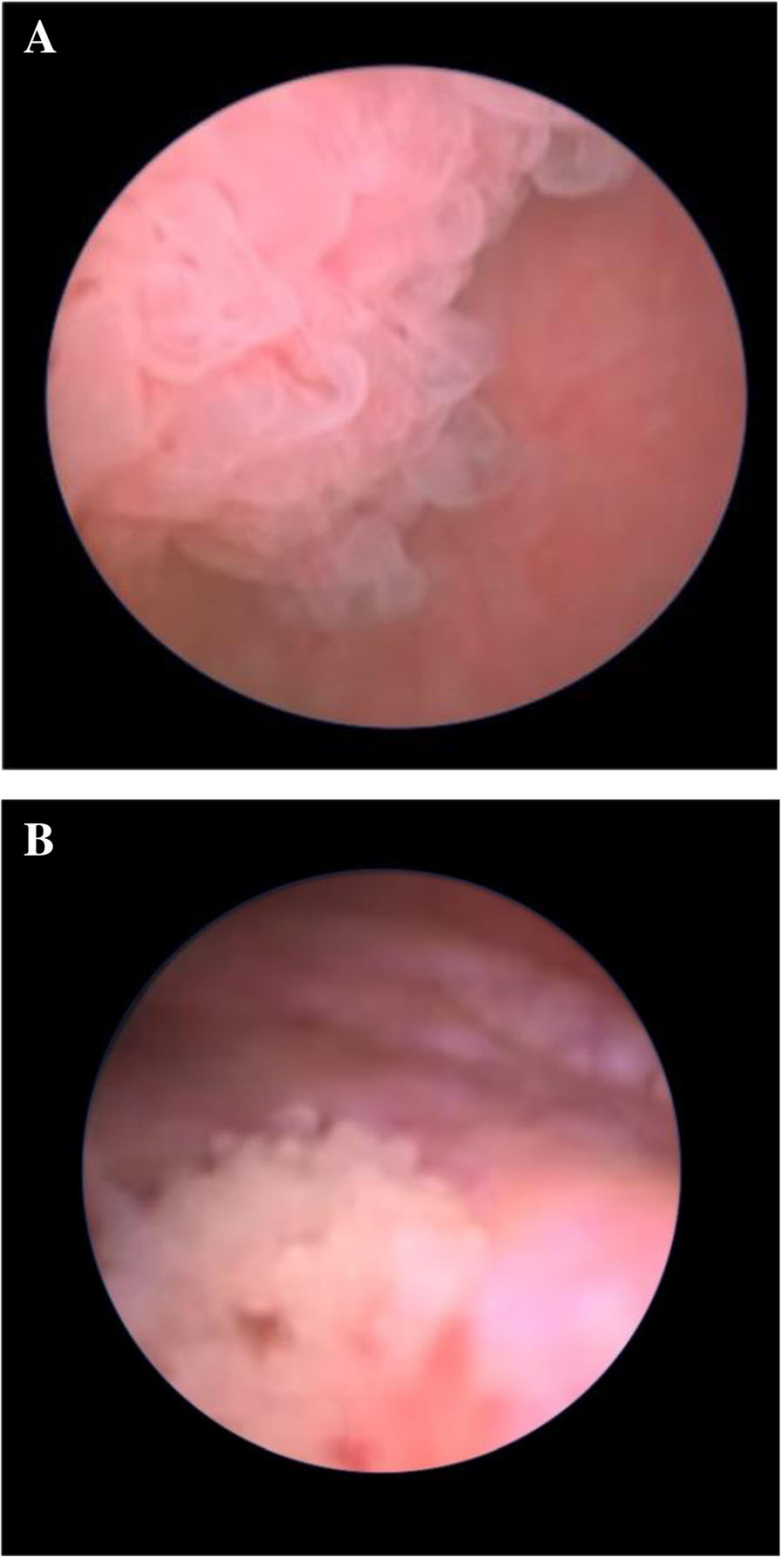
**a** and **b**: Cystoscopic images of multifocal PUNLMP relapse

However, the ultrasonography of bladder performed one year later revealed a dendriform intravesical tumour of the lateral walls and of the bladder bottom. The cystoscopy confirmed the presence of a multifocal relapse of the disease (Figs. 1a and b). The lesions appeared superficial and not infiltrating, sited at the lateral walls and the bladder bottom, with a maximum diameter of 3.5 cm. The histological analysis confirmed the prior diagnosis of PUNLMP.

The computerized tomography with urographic scans (uro-CT) excluded any infiltration of the bladder detrusor muscle and the presence of metastatic disease.

Owing to the clinical history, the histology and the stage of the disease, intra-bladder chemotherapy was adopted. The treatment consisted of a first induction phase comprising mono-weekly intra-bladder instillations of Mitomycin-c (MMC) at a dose of 20 mg for a total of 8 weeks. The cystoscopy performed at the end of the induction phase showed the complete regression of the lesions.

Therefore, maintenance therapy was performed with monthly instillations of MMC at the dose of 20 mg for a total of 6 months. The treatment was well tolerated, without significant complications.

After a month, we performed a close follow-up with renal function, renal and urinary ultrasound, urodynamic evaluation, which was found normal.

Two months later, chemical and cytological urinary tests and cystoscopy were achieved and the random biopsies of the primarily affected areas resulted regular. A TUR was performed during the cystoscopy performed at one year.

Afterward, the child was examined with chemical and cytological urinary tests every three months, besides the renal and urinary ultrasound, the urodynamic evaluation and the cystoscopy were performed every six months in the next two years.

Currently, at five years from the end of the chemotherapy, clinical and instrumental follow-up checks detect the absence of the disease and normal urinary function.

### Discussion and conclusions

PUNLMP is a histopathological entity introduced firstly in the 1998 WHO/ISUP classification.

These neoplasms have a high propensity to local recurrence mostly in the adult population (with a risk of relapse between 40 and 70%), with lower recurrence risk (about 13%) in the young population [6].

The treatment varies widely according to the group of risk. The European Organization for Researches and Treatment of Cancer (EORTC) classifies adult patients based on six different prognostic factors: number of lesions and size of the tumour, previous relapse rate, invasiveness of the lamina propria, concomitant presence of carcinoma in situ and histological grading [7]. Another important prognostic factor is the result of the cystoscopy performed at three months after the TUR [3].

Despite the smallness of the cases, the articles published in the last twenty years suggest overlooking intra-bladder chemotherapy after TUR for the paediatric population with PUNLMP. Although this approach, after one year our patient presented a multifocal recurrence with low-grade and superficial lesions, not infiltrating the lamina propria. Because of the features of the recurrence, the patient belonged to the high-risk group and for the multifocality of the lesions, he would have been subjected to radical cystectomy. Although the lack of standardized recommendations for the treatment of multifocal relapsed PUNLMP of the young age, intra-bladder chemotherapy was suggested for saving organ function [8].

The therapeutic options currently available for adult patients include immunotherapy with the Calmette-Guerin bacillus (BCG) and intra-bladder chemotherapy with MMC, Doxorubicin and Epirubicin [9, 10]. Considering the patient’s age and systemic toxicity related to BCG therapy, we decided to perform treatment with endoscopic intra-bladder MMC instillations. The therapy was well tolerated, with complete remission of the disease at the end of the induction phase.

Then, we made a close follow-up, because of PUNLMP of young age, even if less rarely than in adults, may present an aggressive behavior in terms of recurrence and invasiveness [6, 11, 12].

Concerning the follow-up, although the absence of shared recommendations, the key-examination remains the ultrasound of the urinary tract, which may reduce the frequency of execution of cystoscopy among the paediatric patients [13]. Conversely, cystoscopy remains imperative and should be associated with urinary cytology in the cases of recurrent neoplasms [1, 3, 14].

Intra-bladder MMC instillation seems a safe and effective therapeutic option for paediatric patients with a high-risk relapsed PUNLMP, allowing to achieve the complete remission of disease and the bladder sparing.

Given the paucity of cases and the lack of treatment and follow-up guidelines, it would be necessary to prospectively validate treatment recommendations and share follow-up programs for pediatric patients with PUNLMP.

## Abbreviations

BCG: Calmette-Guerin Bacillus
EORTC: European Organization for Researches and Treatment of Cancer
MMC: Mitomycin-c
PUNLMP: Papillary Urothelial Neoplasm of Low Malignant Potential
TUR: Transurethral resection
Uro-TC: Computerized tomography with urographic scans
WHO/ISUP: World Health Organization / International Society of Urological Pathology
